# SFRmaker and Linesink‐Maker: Rapid Construction of Streamflow Routing Networks from Hydrography Data

**DOI:** 10.1111/gwat.13095

**Published:** 2021-04-08

**Authors:** Andrew T. Leaf, Michael N. Fienen, Howard W. Reeves

**Affiliations:** ^1^ U.S. Geological Survey Upper Midwest Water Science Center 8505 Research Way, Middleton WI 53562 USA; ^2^ U.S. Geological Survey Upper Midwest Water Science Center, Michigan 5840 Enterprise Drive, Lansing MI 48911 USA

## Abstract

Groundwater models have evolved to encompass more aspects of the water cycle, but the incorporation of realistic boundary conditions representing surface water remains time‐consuming and error‐prone. We present two Python packages that robustly automate this process using readily available hydrography data as the primary input. SFRmaker creates input for the MODFLOW SFR package, while Linesink‐maker creates linesink string input for the GFLOW analytic element program. These programs can reduce weeks or even months of manual effort to a few minutes of execution time, and carry the added advantages of reduced potential for error, improved reproducibility and facilitation of step‐wise modeling through reduced dependency on a particular conceptual model or discretization. Two real‐world examples at the county to multi‐state scales are presented.

## Introduction

Increasingly pressing and often complex water resources challenges require groundwater models that more holistically consider the integrated water cycle, and that can be built and updated on faster timeframes. While more advanced boundary condition formulations such as the Streamflow Routing (SFR) Package for MODFLOW (Prudic et al. 2004; Niswonger and Prudic 2005; Langevin et al. 2017) have long been available, they are often underutilized, presumably due to the complexity of input requirements (Anderson et al. 2015). Preparation of SFR input requires mapping linear stream features to a finite‐difference grid, and populating each resulting stream reach with attribute properties. This process can be arduous and error‐prone, requiring many geoprocessing operations or even extensive hand‐digitizing of features. The number and complexity of operations presents a fundamental challenge to scientific reproducibility (e.g., Peng 2011) and step‐wise modeling (Haitjema 1995).

Analytic element programs such as GFLOW (Haitjema 1995) provide an efficient, rapid, and flexible alternative to finite‐differences that facilitates building and refining models iteratively in a step‐wise fashion. A key feature of GFLOW for simulating groundwater/surface water interactions is its built‐in streamflow routing model analogous to the MODFLOW SFR package (Haitjema 1995; Mitchell‐Bruker and Haitjema 1996). However, even GFLOW models require hand‐digitizing and checking of the linesinks that represent streams, a process that can take weeks for regional models with high stream densities.

Construction of model stream networks is often accomplished using mapped hydrography in a geographic information system (GIS) file format, either as a guide for hand‐digitizing or directly as input to geoprocessing routines that map the data to a finite‐difference grid. Hydrography vector data are widely available in datasets such as NHDPlus (McKay et al. 2012; Moore et al. 2019), or can alternatively be generated from a smoothed digital elevation model (DEM) using flow accumulation methods (e.g., Gardner et al. 2018; Ng et al. 2018). The latter approach is best suited to montane areas where topographic flow paths toward streams are well defined, but is limited by the vertical and horizontal accuracy of the DEM, and may still require manual intervention in flat, low‐lying areas (Gardner et al. 2018).

In recent years, open source software tools to automate the translation of hydrography data to model input have become readily available and easy to use. These include Python packages for working with MODFLOW files (Bakker et al. 2016), GIS file formats and geoprocessing (Gillies 2020a, 2020b, 2020c, 2020d), and coordinate transformations (Snow et al. 2020); as well as software development tools that facilitate collaborative version control (e.g., Git; https://git‐scm.com/ and GitHub; https://github.com/), automated testing (e.g., pytest; https://pytest.org), continuous integration, and online documentation (e.g., sphinx; https://www.sphinx‐doc.org/); and accessible tutorials that show domain scientists how to use them (e.g., https://nsls‐ii.github.io/scientific‐python‐cookiecutter).

This paper describes an automated approach to rapidly translate hydrography data into numerical model input. Two open‐source computer programs written in the Python language and distributed as easily installable packages are presented. SFRmaker creates input for the MODFLOW‐2005 (Harbaugh 2005) or MODFLOW‐6 (Langevin et al. 2017) SFR packages; Linesink‐maker creates input for the analytic element program GFLOW (Haitjema 1995). These programs can reduce what previously required weeks or even months of effort to a few minutes of execution time. To our knowledge, no similar programs exist currently. In developing and maintaining these programs, we have sought to leverage open‐source software tools and best practices (e.g., Wilson et al. 2014; https://nsls‐ii.github.io/scientific‐python‐cookiecutter) to improve the robustness, quality, transparency, and maintainability of the code. By robustly automating a burdensome and error‐prone task, we hope that these tools can both improve the quality of boundary conditions in groundwater models, and allow modelers to focus more on the most important aspects of their problems. This paper includes a brief description of the two programs and an example for each.

## Software Implementation

Both SFRmaker and Linesink‐maker are implemented as Python packages that work on Linux, OSX or Windows. The versions of the code documented in this paper are available as USGS software releases (Leaf et al. 2021; Leaf 2021); current development versions that incorporate bug fixes and other improvements are available through GitHub (https://github.com/usgs/sfrmaker; https://github.com/usgs/linesink‐maker) or the Python Package index (PyPI). Note that both programs have software dependencies that must be installed prior to their use. Detailed instructions on how to install the dependencies and SFRmaker and Linesink‐maker are available in their online documentation (https://usgs.github.io/sfrmaker/; https://usgs.github.io/linesink‐maker/). Like any Python package, SFRmaker and Linesink‐maker are made up of objects that can be imported into a Python session and therefore used within scripts or other Python code. This allows for many different use cases, such as adding specified flows to an existing SFR package, or incorporating setup of the SFR package into an automated workflow for building a complete groundwater model. Use of SFRmaker and Linesink‐maker does not require extensive knowledge of Python, however. For the typical use case of creating a MODFLOW SFR package or GFLOW linesink network, input can be specified in a configuration file, which is then read by a simple, generic script that makes the necessary method calls to do the processing. The examples in this paper will focus on use of the configuration file; additional details on scripting with SFRmaker and Linesink‐maker are provided in the online documentation.

## Methods

The core function of both SFRmaker and Linesink‐maker is to reformat hydrography vector data into model input describing head‐dependent flux boundaries representing streams, where groundwater/surface water interactions are limited by the availability of water in the stream channel. Linesink‐maker also creates resistance linesinks that represent drainage lakes within the stream network and seepage lakes that are not connected to the stream network (e.g., Born et al. 1979). SFRmaker accepts either custom hydrography (as shown below in the MERAS example) or native NHDPlus (version 2; McKay et al. 2012). Currently Linesink‐maker only works with NHDPlus version 2, although it could be easily adapted to work with other hydrography.

At the lowest level, hydrography vector data consist of sequences of points or vertices defining curves or arcs that represent a section of stream; often between two confluences. Following the terminology of the Shapely Python package (Gillies 2020d), we will refer to these arcs as *linestrings*. A hydrography dataset consists of a collection of linestrings and associated attribute information, often serialized in the shapefile format (ESRI 1998), which is the typical input format to SFRmaker and Linesink‐maker. We use the NHDPlus term *flowlines* to refer more broadly to a hydrography dataset that includes both linestrings and their attributes. The basic process to create model streamflow routing input, and the software packages used, are summarized below.

### Discretization of Hydrography

**Figure 1:**
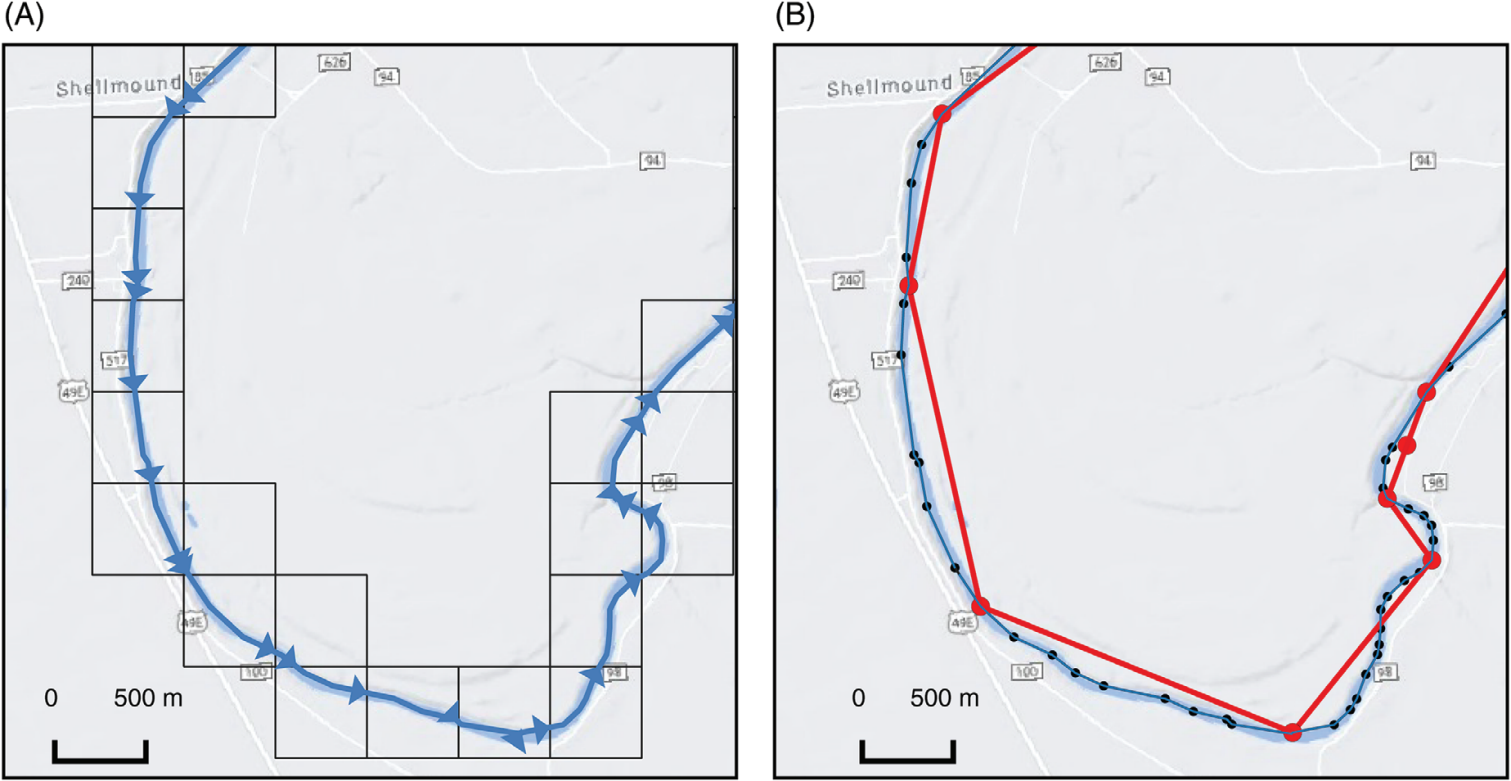
Discretization applied by (A) SFRmaker and (B) Linesink‐maker. The original hydrography are shown as the black dotted lines in (B). SFRmaker subdivides flowlines at the model cell boundaries. Each blue arrow in (A) represents an SFR reach. Linesink‐maker removes vertices from the original flowlines until a minimum number is achieved that maintains a distance tolerance from the original line (250 m for the red line in [B]). Each segment of the red line in (B) represents a linesink element.

In both SFRmaker and Linesink‐maker, the source hydrography data are read into a Pandas DataFrame (The Pandas Development Team 2020) using the Fiona package (Gillies 2020a), reprojected to the model coordinate reference system (CRS) if necessary, and mapped to the model discretization. SFRmaker subdivides linestrings into reaches that each coincide with a single finite‐difference cell (Figure 1). The process of mapping intersections and attributes between two sets of geometric features (a spatial join) requires that each potential intersection be evaluated. SFRmaker uses an R‐tree spatial index (Gillies 2020c) to speed this process. In analytic element approaches such as GFLOW, streams are discretized into *linesinks* defined by pairs of points, which are grouped consecutively into *linesink strings* analogous to linestrings. Each linesink represents an equation that is superimposed on the composite analytic solution for the model. While the time required to solve an individual GFLOW model varies depending on the degree of nonlinearity and other factors, in practice, 3–4,000 linesink equations represents a practical upper limit of what is solvable in the current version. Representing a linestring in the source hydrography requires *n* − 1 linesinks, where *n* is the number of vertices in the linestring. In humid regions with high stream densities, this might result in tens of thousands of equations for a county‐scale model. To reduce this number, Linesink‐maker uses the feature‐simplification algorithm in Shapely (Gillies 2020d) to minimize the number vertices subject to a distance tolerance (Figure 1). Different levels of tolerance can be assigned to a detailed nearfield area of interest, a midfield containing routed resistance linesinks, and a coarsely discretized farfield that forms a perimeter boundary of zero‐resistance linesinks (e.g., Haitjema 2005).

In GFLOW, lakes are represented with (linear) linesink elements along their shorelines, and can therefore be readily produced using the same methods as streams. Linesink‐maker uses the NHDPlus waterbody dataset (McKay et al. 2012) to produce both drainage lakes that are connected to the stream network and seepage lakes that do not have inlets or outlets. In NHDPlus, drainage lakes are represented both as polygon features in the waterbody dataset and linear features in the flowlines dataset that connect inlets and outlets. Flowlines representing lakes are cross‐referenced with waterbody polygon features using the COMID or Common Identifier numbers (McKay et al. 2012). Linesink‐maker uses this cross‐referencing to replace drainage lake flowlines with linesinks that wrap around the lake perimeter. SFRmaker only represents drainage lakes implicitly based on their flowline representations. Explicit creation of any lake input for MODFLOW is beyond the scope of SFRmaker.

The result of the discretization step is a second DataFrame with a row for each SFR package reach or GFLOW linesink, with an identifier column mapping each reach or linestring back to the original feature that it came from (e.g., in NHDPlus, the COMID). In SFRmaker, the original flowlines form the basis of segments (sequential groupings of reaches), which are numbered to strictly increase in the downstream direction (Prudic et al. 2004). Each row in the DataFrame has a Shapely LineString object representing the geometry associated with that reach or linesink.

### Specification of Routing

Routing information in the original hydrography input is then used to define a routing column with the identifier of the next reach or linestring downstream. Following the convention of the MODFLOW SFR package, a value of zero indicates an outlet condition (flow leaving the model). SFRmaker only supports routing to a single downstream reach. If multiple downstream connections are supplied, the connection with the lowest starting elevation or the first connection listed (in lieu of elevation information) is chosen. Linesink‐maker does not handle routing explicitly, as this is performed by the GFLOW GUI based on linesink proximity and elevations.

### Streambed Elevations

**Figure 2:**
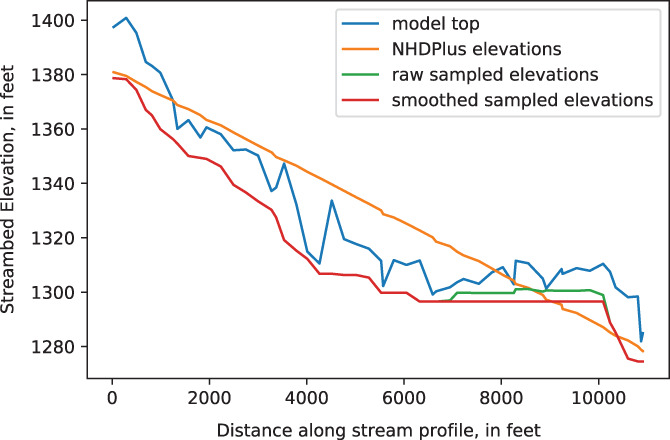
Comparison of elevation profiles along a stream. The model top (mean DEM elevation within each model cell), is jagged along the stream profile due to topographic variability within the model cells and mismatch between the hydrography linestring and the DEM. The NHDPlus elevations are representative of the stream channel at the segment ends, but float substantially above the channel in the interior, due to the nonlinear channel profile. In this case, the minimum elevations sampled from the DEM are mostly smooth, but still contain a rise near the end of the segment. The smoothed elevations do not rise, but may still float above the actual channel in some places, as the true channel elevation is not known everywhere.

Starting and ending streambed elevations for each flowline are read from the original hydrography data and distributed to any intervening reaches by linear interpolation. This is the default method in SFRmaker, and the only method for assigning streambed elevations in Linesink‐maker. Alternatively, SFRmaker can sample minimum elevations from a DEM, within a buffer polygon surrounding the linestring for each reach (Perry 2020; Gillies 2020d). Minimum DEM elevations are assumed to best reflect the actual stream channel elevations. However, because of mismatch between the hydrography lines and DEM, and topographic variability at scales finer than the DEM resolution, sampled minimum elevations do not always reflect the channel. This can be seen in sampled elevations that rise in the downstream direction. SFRmaker addresses this by smoothing the sampled elevations so that no elevation can be higher than the minimum elevation encountered upstream (Figure 2).

### Lake Linesink Elevations

Unlike drainage lakes, seepage lakes in the NHDPlus dataset do not have elevations. Linesink‐maker obtains elevations for seepage lakes by querying the National Map Elevation Point Query Service (EPQS; https://ned.usgs.gov/epqs/) at point locations within each lake. An internet connection is required for this to work; otherwise, elevation values of 0 are returned, and must subsequently be adjusted by the user.

### Stream Widths

The width parameter is important for accurately representing groundwater/surface water interactions when there is resistance to flow between a surface water body and the aquifer (see, e.g., Prudic et al. 2004; Haitjema 2005). Both SFRmaker and Linesink‐maker estimate stream channel width using an empirical relationship with arbolate sum (the total length of upstream drainage; Feinstein et al. 2010; Moore et al. 2019). Linesink‐maker computes width parameters for lakes following the methodology of Haitjema (2005).

### Handling of Colocated SFR Reaches

**Figure 3:**
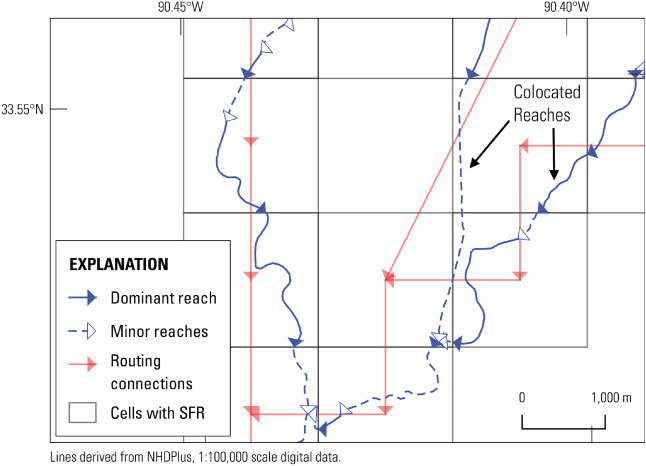
Illustration of reach consolidation for colocated streams. The blue lines represent the original hydrography; the red lines show routing connections between model cell centers. One SFR reach per model cell is retained (solid blue lines with blue arrows; the dashed blue lines with white arrows represent colocated reaches that were consolidated). The total conductance of all reaches is summed for each model cell, and applied to the single (dominant) reach with the highest arbolate sum. Conductances for the remaining (minor) reaches are either set to zero, or these reaches are removed entirely from the SFR package. When minor reaches are removed, routing connections are reorganized to connect the remaining dominant reaches. This process is illustrated by the cell near the upper right corner with the “colocated reaches” label. Within this cell, the reach in the right tributary (solid line) has a larger arbolate sum and is retained as the dominant reach. The reach in the left tributary (dashed line) is removed. The next dominant reaches upstream and downstream of the removed reach are then connected to maintain continuity in the stream network, as illustrated by the red line that cuts diagonally through the cell.

Large model cell sizes and a high density of streams can result in SFR reaches that are colocated within a model cell (Figure 3). While the SFR package can compute flows between colocated reaches and the groundwater flow solution, a high number of colocated reaches can slow model solution times and result in an unnecessarily large SFR package that is slow to read and write. To address this issue, SFRmaker can consolidate colocated reaches by applying the sum of SFR reach conductances in a cell to a dominant reach, and setting the conductances of other minor reaches to zero. Conductance is computed as the product of reach length, width, and streambed vertical hydraulic conductivity (*K*_*v*_) divided by the streambed thickness (e.g., Prudic et al. 2004); SFRmaker adjusts conductance through the *K*_*v*_ term. Minor reaches with zero conductance can be left in the SFR package (default) or removed, as shown in Figure 3.

### Other Input

Finally, the remaining input parameters are populated. By default, SFRmaker assumes global streambed thickness and vertical hydraulic conductivity values of 1 (prior to any reach consolidation), and that stream stage will be estimated using Manning's equation (e.g., icalc = 1, Prudic et al. 2004; Niswonger and Prudic 2005; or status = ACTIVE, Langevin et al. 2017), with a default global roughness value of 0.037 (e.g., Arcement and Schneider 1989), and default minimum slope of 10^−4^. These and other settings can be changed in a scripting context via keyword arguments to the SFRData object, or after running SFRmaker, through modification and subsequent regeneration of the SFR package file from the SFRmaker tabular output. Additional details are available in the SFRmaker online documentation. All parameter options to Linesink‐maker are set through the configuration file, as illustrated in the Medford National Forest example.

### Diagnostics

To validate the SFR package, SFRmaker checks for not a number (Nan) values, valid segment and reach numbering, circular or nonadjacent routing connections, inconsistencies in elevations within the SFR package and with the model grid (if a model is included), colocated reaches, and spurious values of other input parameters such as slope. Results of the checks are recorded in a text file. SFRmaker also produces shapefiles for visualizing the linestrings and finite‐difference cell polygons associated with each stream reach, as well as routing connections, outlet locations where flow is leaving the SFR network, and inlet locations where inflows are specified.

By default, Linesink‐maker checks for and corrects streambed elevation gradients that are flat or run uphill, and reports any Linesinks that cross (a common side‐effect of simplification), so that they can be fixed by the user prior to running GFLOW. The GFLOW GUI itself handles routing internally, and includes features for visualizing routing connections and the conjunctive stream/groundwater‐flow solution (Haitjema 2020).

## SFRmaker Example: An Updated Streamflow Routing Network for the Mississippi Embayment

**Figure 4:**
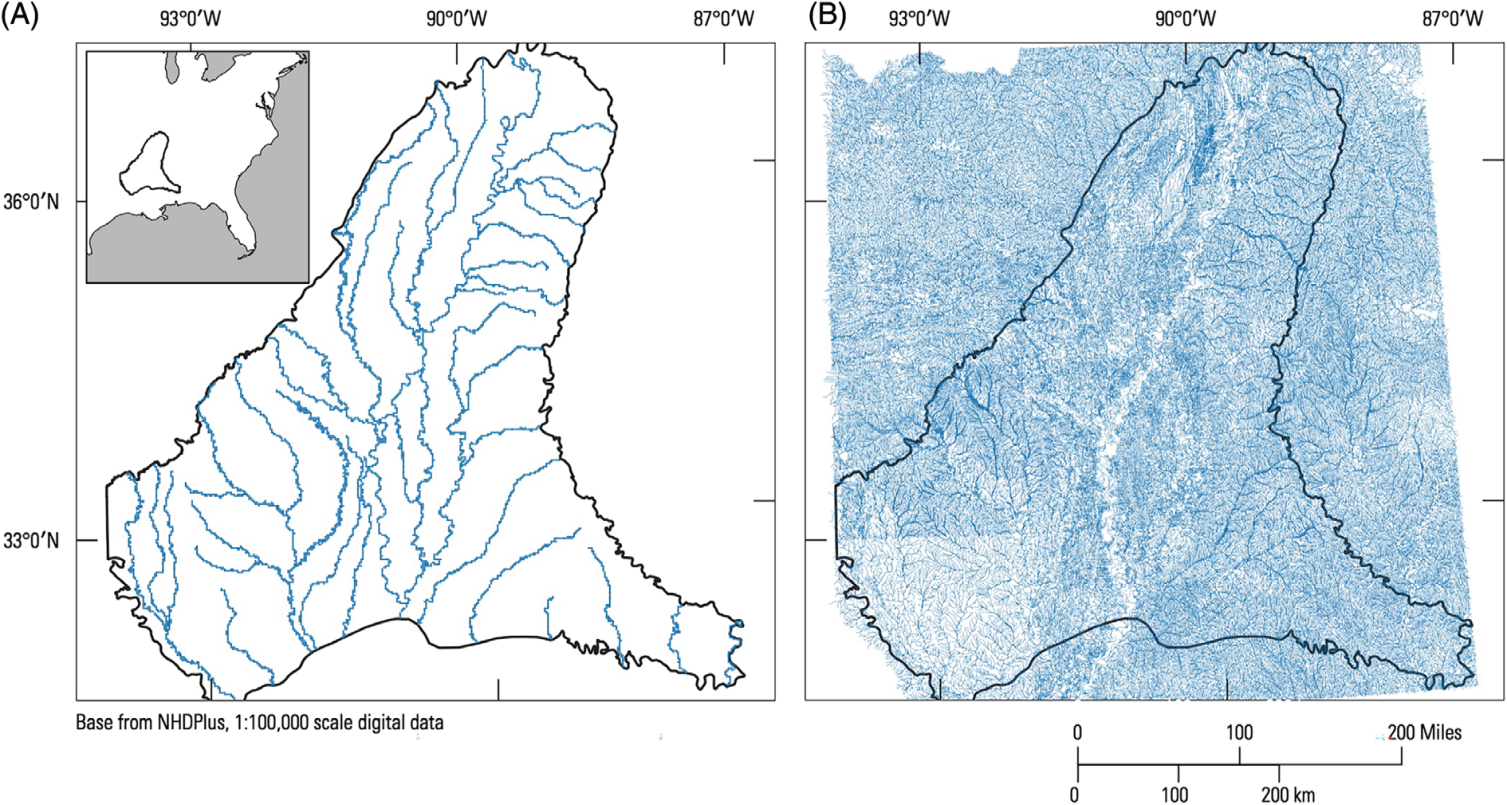
MERAS model extent with streams represented in the MERAS 2 model (A) compared to streams mapped in NHDPlus version 2 (B).

The Mississippi Embayment region is a historical bay of the Gulf of Mexico that extends southward from the present‐day confluence of the Ohio and Mississippi Rivers. Within the Mississippi Embayment, increasing irrigation withdrawals from the Mississippi River alluvial aquifer (MRVA) have led to groundwater level declines, creating concerns about future sustainability (Barlow and Clark 2011; Konikow 2015). Groundwater modeling with MODFLOW is being used to support future management decisions (e.g., Haugh et al. 2020). The thousands of mapped streams within the Mississippi Embayment are a potential source of water to the MRVA in areas where groundwater levels are depressed. However, previous modeling efforts (the Mississippi Embayment Regional Aquifer System or MERAS model; Haugh et al. 2020; Clark and Hart 2009) only included the 43 largest streams (Figure 4A). A key component of ongoing modeling efforts is to expand the number of streams represented.

The configuration file for the Mississippi Embayment example is shown below. All input needed to reproduce the example is available on the SFRmaker GitHub page. The configuration file is specified in the YAML format (yaml.org), which maps key: value pairs similar to a Python dictionary (https://docs.python.org/3/tutorial/datastructures.html). In the SFRmaker configuration file, keys (to the left of the colons) indicate variables or groups of variables.
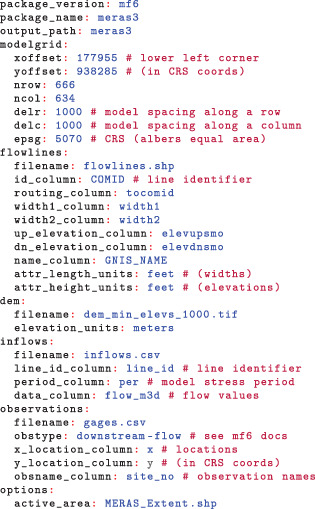


The model itself has a uniform grid spacing of 1,000 m on a cell side, which is aligned with the National Hydrogeologic Grid (Clark et al. 2018). Currently, SFRmaker only supports structured grids. Information on the model grid is specified in the modelgrid: block of the configuration file. The xoffset: and yoffset: arguments are the location of the lower left corner of the model grid, in units of the CRS defined by the EPSG code (https://epsg.org; typically meters). As this is a uniform grid, only scalar values are provided for the row and column spacing. Variable row and column spacings can be specified using the list notation in YAML, or more simply by loading a MODFLOW model into a Python script using the FloPy package (Bakker et al. 2016), as shown in the examples in the SFRmaker online documentation. SFRmaker does not require a model as input, but a model can optionally be specified in the configuration file with the model: and simulation: blocks (the latter is only needed for MODFLOW‐6 models):
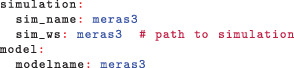


An advantage of specifying a model is that SFRmaker will automatically assign stream reaches to the correct layer, based on the layer top and bottom elevations, and account for inactive cells. If the model has correct georeference information in the namefile header as assigned by FloPy (Bakker et al. 2016), the modelgrid: block is not needed. An optional active_area: key allows for specification of a geographic area for generation of the SFR package, which may or may not coincide with the active area of the model. Otherwise, SFR input will be generated for the active extent of the model grid.

The MERAS example shows how custom hydrography can be supplied to SFRmaker by explicitly specifying a shapefile and the attribute field names in the input. For this case, linestring vector features representing mapped hydrography were obtained from NHDPlus version 2 (McKay et al. 2012). The MERAS model extent shown in Figure 4 overlaps eight major drainage basins that include more than 1 million linestring features. To achieve a tractable workflow, the original NHDPlus data were clipped to the MERAS footprint and flowlines with a arbolate sum of 20 km or greater were retained. This smaller dataset was saved to a shapefile (*flowlines.shp*) along with the relevant attribute information. Alternatively, NHDPlus data can be used directly by simply giving SFRmaker the path to the root‐level folder for each drainage basin (assuming the same file structure as NHDPlus). With this option, only filepaths are needed, as the locations of the attribute data are already understood by SFRmaker. A list can be used to include more than one drainage basin.

While the MERAS and NHDPlus hydrography include elevations, this may not always be the case, or more accurate elevations may be available from a DEM. If a dem: block is specified, SFRmaker will sample the DEM to the stream reaches as described in the methods section. The default buffer distance is 100 in the units of the projected CRS, or another buffer distance can be specified with an optional buffer_distance: argument.

Many streams enter the Mississippi Embayment with appreciable flow, which must be included in the SFR package to achieve a realistic mass balance. The inflows: block allows for specification of such flows by model stress period (SFRmaker currently does not do any resampling), in a comma‐separated‐variable (CSV) format. Similarly, an observations: block allows observation site locations to be specified in the CRS of the model grid, or using identifiers corresponding to flowlines in the input hydrography. With this information, SFRmaker will generate input to the Gage Package (Prudic et al. 2004) or MODFLOW‐6 observation utility (Langevin et al. 2017).

The above configuration file can be used to generate an SFR package with the following Python code:


from sfrmaker import SFRData

sfrdata = SFRData.from_yaml

('input.yml')



**Figure 5:**
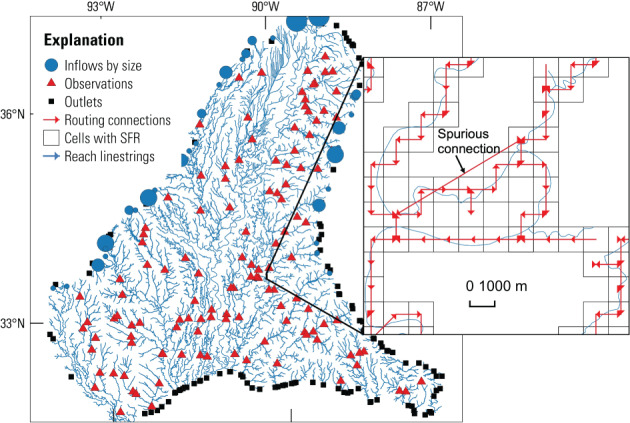
Shapefile output showing the updated MERAS SFR package. Visualization of routing connections is illustrated in the map inset, where a connection crossing several cells indicates an error in the input hydrography routing attributes. The fix for this error is to edit the routing connections in the source hydrography and then re‐run SFRmaker.

This will produce an sfr package for MODFLOW‐6, CSV table representations of the SFR input, and shapefiles for visualizing the SFR package. Figure 5 shows the shapefile output for the MERAS example, which reveals an error in the input hydrography routing attributes. While the diagnostic output reports any reach connections longer than 1.25 times the hypotenuse of their cells (the length of a diagonal connection between two cells, where one cell is 50% longer on a side), with reach consolidation (Figure 3), there may be many long connections reported, making true errors more difficult to distinguish. The fix for this error is to edit the routing connections in the source hydrography and then re‐run SFRmaker.

## Linesink‐Maker Example: Medford Unit of the Chequamegon National Forest

The example is based on a published GFLOW model of the Medford Unit of the Chequamegon‐Nicolet National Forest in northern Wisconsin. (Bradbury et al. 2018). The purpose of the model was to improve understanding of the groundwater flow system and groundwater/surface water interactions within the forest unit, especially for management of groundwater dependent ecosystems. The configuration file for the Medford example is shown below. All input needed to reproduce the example is available on the Linesink‐maker GitHub page.
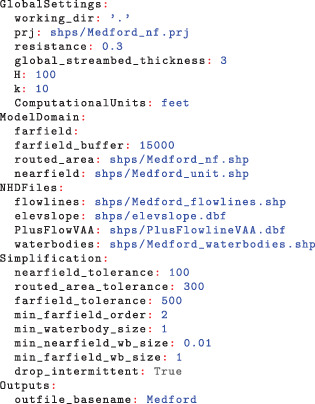


The GlobalSettings: block in the configuration file contains settings that apply to the whole model. The resistance:, global_streambed_thickness:, H: (representative aquifer thickness), and k: (representative aquifer hydraulic conductivity) are all used to compute the characteristic leakage length (*λ*) needed for estimating an appropriate width parameter for lakes (Haitjema 2005), and are given in the model length units (ComputationalUnits:). working_dir: specifies the location where output from Linesink‐maker will be written; prj: is a file path to a projection file containing a well‐known text (WKT; http://www.opengis.net/doc/is/wkt‐crs/2.0.6l) representation of the projected CRS for the model.

The ModelDomain: block allows specification of different areas of model refinement. With the nearfield: key, the user can specify a polygon shapefile defining the primary area of focus for the model. With the optional routed_area: key, another polygon can be supplied to define the extent of the model stream network of routed, resistance linesinks (see Haitjema 1995). Finally, the outer extent of the model can be defined using a polygon shapefile with the farfield: key, or alternatively, as a buffer distance around the nearfield polygon with the farfield_buffer: key. The area between the nearfield polygon (or optionally, the routed area polygon) and the farfield extent is then populated with zero‐resistance linesinks that form a perimeter boundary condition (see Haitjema 1995).

Source hydrography input are defined in the NHDFiles: block as shown. As of this paper, Linesink‐maker only works with NHDPlus data. The Simplification: block controls how the hydrography input are discretized, and which features are retained. For example, a nearfield_tolerance: value of 100 m means that the simplification of the original flowlines will be limited by the constraint that the simplified lines do not deviate from the original lines by more than this distance. With the min_farfield_order: key, lower‐order streams can be excluded from the farfield linesinks (a value of 2 means that first‐order streams are excluded). The min_waterbody_size:, min_nearfield_wb_size: and min_farfield_wb_size: keys control the minimum size for the waterbodies that are included in the routed, nearfield and farfield areas of the model (in square km). Finally, with the drop_intermittent: key, streams classified as “intermittent” in NHDPlus can be excluded from the routed part of the model outside of the model nearfield. By default, all streams are included in the nearfield.

The above configuration file can be used in the following script to generate a linesink string (LSS) XML file of the stream network that can be imported into GFLOW (version 2.2 or later; Haitjema 2020).


import lsmaker

ls = lsmaker.LinesinkData('Medford_lines.yml')

ls.make_linesinks()



**Figure 6:**
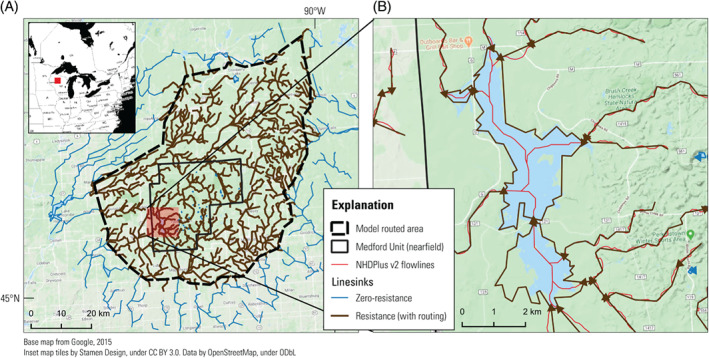
(A) Linesinks produced by Linesink‐maker for the Medford Unit of the Chequamegon‐Nicolet National Forest. A distance tolerance between the simplified linesinks and original hydrography controls the level of detail in the stream network. Linesinks within the forest unit were created at the highest level of detail (100 m distance tolerance). A buffer of routed resistance linesinks discretized at a 300 m tolerance surrounds the forest unit, to allow for accurate simulation of hydraulic divides between competing sinks and stream (base) flows into the forest unit. Coarsely discretized (500 m tolerance) zero‐resistance linesinks create a perimeter boundary condition for the solution. (B) The conversion of flowlines (red) into a drainage lake represented by routed resistance linesinks around its perimeter. Linesink‐maker makes small adjustments to the end elevations of drainage lake tributaries to ensure proper routing in the GFLOW GUI.

A shapefile representation of the linesinks is also produced, along with additional shapefiles of the source hydrography merged and clipped to the model area. The resulting linesinks are shown in Figure 6.

## Discussion and Conclusions

The examples presented show how SFRmaker and Linesink‐maker can produce model input for large stream networks, which could take weeks or even months to produce manually, in a matter of minutes. In addition, earlier versions of SFRmaker and Linesink‐maker have been used successfully to create stream networks for numerous published models (e.g., Leaf et al. 2015; Masterson et al. 2016; Bradbury et al. 2018; Davis and Long 2018; Fienen et al. 2018; Fehling et al. 2018a, 2018b; Haserodt et al. 2019; Parsen et al. 2019; Feinstein et al. 2020). Linesink‐maker is also routinely used by the Wisconsin Rural Water Association to improve wellhead protection plans that would otherwise not have the budget for a groundwater flow model (A. Aslesen, Source Water Specialist, written communication, 2020). Advantages of the automated approach implemented in SFRmaker and Linesink‐maker include:

- Reduced time and labor required to make a groundwater model.
- Reduced potential for human error in the creation of streamflow routing input. If a bug is identified in the code, a single fix can be applied to all subsequent applications. Continuous integration testing helps verify that the code is working as intended.
- Improved reproducibility.
- Reduced dependence on a single model grid or conceptual model. The ability to robustly generate an SFR package in a few minutes makes it easier to change the stream network or other aspects of the model structure, which facilitates step‐wise modeling.

Despite these advantages, the quality of groundwater models still ultimately depends on *hydrosense* (Hunt and Zheng 2012); it is up to the modeler to check the results and modify them as needed.

## Future Work

Development of SFRmaker and Linesink‐maker has been primarily driven by project needs. As a result, these packages do not provide comprehensive support for the features available in the MODFLOW SFR package or GFLOW linesinks. For example, advanced SFR input options including unsaturated flow beneath streams, nonrectangular channel geometries, and diversions are not currently supported. Support for unstructured grids is not yet implemented, although the code is designed to allow for that extension in the future. Linesink‐maker currently does not support creation of linesink lakes with a separate stage solution. The object‐oriented design of these packages and collaborative version control can facilitate the addition of these and other features, such as input to other modeling codes. The authors welcome contributions to these packages; more information about this can be found in their online documentation.

## Authors' Note

The authors do not have any conflicts of interest or financial disclosures to report.

##  

 
